# Importance of extending the use of polymerase chain reaction in the diagnosis of venereal syphilis in a blood transfusion center in Burkina Faso, West Africa

**DOI:** 10.11604/pamj.2014.18.56.3850

**Published:** 2014-05-15

**Authors:** Abibou Simpore, Cyrille Bisseye, Bolni Marius Nagalo, Mahamoudou Sanou, Yacoub Nébié, Laure Stella Ghoma-Linguissi, Honorine Dahourou, Boukary Sawadogo, Florencia Djigma, Siaka Ouattara, Virginio Pietra, Jason Nichol, Jacques Simpore

**Affiliations:** 1 Pietro Annigoni Biomolecular Research Center (CERBA)/ (LABIOGENE), University of Ouagadougou, Burkina Faso; 2 National Blood Transfusion Center, Ouagadougou, Burkina Faso; 3 Laboratory of Molecular and Cellular Biology (LABMC), University of Sciences and Techniques of Masuku (USTM), Franceville, Gabon; 4 Endicott College, College of Arts and Sciences, Beverly, USA; 5 Healthcare Unit Training, (UFR-SDS), University of Ouagadougou, Ouagadougou, Burkina Faso; 6 Marlboro College, Vermont, USA

**Keywords:** Treponema pallidum, diagnosis, PCR, RPR, CMIA

## Abstract

**Introduction:**

Due to the existence of a variety of types of non-venereal syphilis caused by the related *T. pallidum*, regular serological testing such as Rapid Plasma Reagin (RPR) and Chemiluminescent Microparticle Immunoassay Technique (CMIA) are often unable to differentiate venereal syphilis from the non- venereal one, hence, the interest in the use of molecular biology testing for a confirmation diagnosis of syphilis caused by *Treponema pallidum* subspecies *pallidum*.

**Objective:**

The study is designed to assess the effectiveness of PCR testing and serological methods in the diagnosis of *Treponema pallidum subsp pallidum* among blood donors in Burkina Faso.

**Methodes:**

The study included 6375 samples of volunteer blood donors from the regional blood transfusion center of Ouagadougou (CRTS/O). Among samples, 183 positive and 59 negative in RPR were analyzed to detect antibodies anti-*T. pallidum subsp pallidum* with a immunoassay method (CMIA) and were confirmed using the Polymerase Chain Reaction testing.

**Results:**

In RPR, we obtained a prevalence rate of 2.9% (183/6375) for treponematosis. From the 183 RPR+ specimen, 108 (59%) were found CMIA+ and 11 (6%) were confirmed PCR+. While the 59 pattern RPR-; 31 (52.5%) were CMIA + including 3 (5.1%) tested PCR+. Seventy-five (75) samples RPR + /CMIA-; 2 (2.7%) were confirmed positive by PCR. All 28 samples RPR-/CMIA- were confirmed negative by PCR.

**Conclusion:**

PCR testing confirmed a low distribution of *T. pallidum subsp pallidum* in comparison to serological methods. Cross-reactions, existence of non-venereal treponemal or immunological scars could account for the discrepancy between the results obtained.

## Introduction

Despite significant scientific advances in the diagnosis of blood-borne and sexually transmitted diseases, the Human Immunodeficiency Virus (HIV), viral hepatitis and syphilis continue to be major public health issues in Sub Saharan Africa [1, 2]. Although the existence of a wide range of serological testing (such as *Treponema pallidum* haemagglutination test (TPHA), Fluorescence *Treponema* antibody (FTA), Rapid Plasma Reagin (RPR), Enzyme-linked immunosorbent assay (ELISA)...) has drastically improved the routine diagnosis of syphilis [3], the majority of these methods is limited because of their low specificity (non-venereal Treponema, cross-reactions, seroconversion...) and laboratory mistakes [4–6]. Blood transfusion safety begins with the pre-donation interview that eliminates high-risk blood donors, particularly those who have had risky behavior in the last three months prior to donation [7]. Then, to eliminate all the infected blood donations, the laboratory performs a series of blood screenings for infectious diseases markers.

In Burkina Faso, the prevalence of syphilis is high and variable: 1.7%, 5.7% and 2.1% respectively for blood donors of Ouagadougou, the prison population and four regional blood transfusion centers [8–10]. The high prevalence and other markers detected in blood donors are corroborated by the work of Nagalo and al. [10]: anti-HIV (1.8%), HBsAg (13.4%), and anti -HCV (6.3%). There is an urgent need to shift to new and effective diagnosis methods that would prevent large numbers of patients from post- transfusion complications. This study is a description of the interest in the use of PCR to diagnose venereal syphilis in a sample of volunteer non-remunerated blood donors in Burkina Faso.

## Methods

**Samples collection:** Two hundred forty-two (242) blood donors were included in this study, from August 2011 to December 2012, with blood donors ranging in age from 17 to 60 and coming from different occupational categories. Reactive or non-reactive RPR plasmas were stored at -20°C for the detection of *Treponema pallidum* genome.

**Serological analysis:** The presence of antibodies to *Treponema pallidum* was detected using Rapid Plasma Reagin testing (RPR) (FUTURA SYSTEM, Roma, Italia) and ARCHITECT Syphilis TP assay (Syphilis TP; Abbott Japan, Tokyo, Japan) a two-step sandwich chemiluminescent microparticle immunoassay (CMIA: ARCHITECT-i100SR-ABBOTT).

Hepatitis B surface antigen (HBsAg), antibodies to HCV and HIV types 1 and 2 were screened using a fourth generation ELISA (ARCHITECT-i100SR-ABBOTT, Santa Clara, California, United States of America). All the reactive samples for HIV, HBsAg, and HCV were re-tested for confirmation using a second enzyme-linked immunosorbent assay (Bio-Rad, Marnes la Coquette, France). A result is considered positive if both the first and second tests were positive.

***Treponema pallidum pallidum*** **DNA extraction and Amplification:** DNA extraction was performed using the DNA-Sorb-B kit (Sacace Biotechnologies, Como, Italy) following the manufacturer′s instructions. The extracted DNA is used for PCR amplification using the *Treponema pallidum* 273/668IC PCR kit with electrophoretic Detection (Sacace Biotechnologies, Como, Italy).

**Statistical Analysis:** Data were analyzed using SPSS 17 .0 and Epi Info 3.5.1 softwares. The Chi-square test was used for comparisons. P-values

## Results

**Sociodemographic characteristics of blood donors:** Two hundred forty-two (242) donors were included in this study. The subjects? average age was 26.9 years (range of 17 to 60 years). Donors were mostly from 21-30 year age group (61.6%); 81.0% were males and 71.5% were first-time donors. The majority of donors were recruited in urban areas (79.8%). Repeat donors were mainly from the age group 21-30 (32.2%) (Table 1).

**Table 1:** Show the socio-demographic characteristics of VNRBD

Characteristics		N (%)	Type of donors	
			First-time donors	Repeat donors
Gender	Female	46 (19. 0%)	39 (84.8%)	7 (15.2%)
	Male	196 (81. 0%)	134 (68.4%)	62 (31.6%)
Years	17-20	41 (16.9%)	31 (75.6%)	10 (24.4%)
	21-30	149 (61.6%)	101 (67.8%)	48 (32.2%)
	31-40	29 (12.0%)	23 (79.3%)	6 (20.7%)
	41-60	23 (9.5%)	18 (78.3%)	5 (21.7%)
Place of blood collection	Urban areas	193 (79.8%)	137 (71.0%)	56 (29.0%)
	Rural areas	49 (20.2%)	36 (73.5%)	13 (26.5%)
Total		242	173 (71.5%)	69 (28.5%)

VNRBD****, volunteer non remunerated blood donor

**Syphilis serology and viral co-infections in blood donors:** Of the 242 donors tested, 183 plasmas (75.6%) were reactive to RPR, and only 139 (57.4%) were reactive to CMIA. The difference in the detection of *T. pallidum pallidum* was significant between RPR and CMIA (p< 0,001). HBsAg, anti-HCV, and anti-HIV antibodies were detected in respectively 24 (9.9%), 28 (11.6%), and 3 (1.2%) blood donors.

**PCR confirmation of anti-Treponema:** Diagnosis results of *T. pallidum* PCR showed that 170/183 (92.9%) and 125/139 (89.9%) respectively detected by RPR and CMIA were negative by PCR (Table 2). The DNA of *T. pallidum* subspecies *pallidum* was found in 16 out of 242 (6.6%) blood donors. Out of 108 samples detected positive using serological methods, 97 (89.8%) were negative by PCR for *T. pallidum*, and only 11/108 (10.2%) were positive.

**Table 2:** Results of the serological screening in the detection of T. pallidum versus PCR

	Number tested	PCR-	PCR +
RPR- & CMIA-	28	28/28 (100.0%)	0
RPR- & CMIA +	31	28/31 (90.3%)	3/31 (9.7%)
RPR+ & CMIA-	75	73/75 (97.3%)	2/75 (2.7%)
RPR+ & CMIA +	108	97/108 (89.8%)	11/108 (10.2%)
Total	242	226 /242 (93.4%)	16/242 (6.6%)

## Discussion

Molecular amplification techniques are struggling to establish themselves in a hostile environment of the African continent, which is marked by economic difficulties, a lack of adequate technical equipment, and a shortage of qualified and competent personnel. However, the high prevalence of blood-borne infections and sexually transmitted diseases has drastically limited the socio-economic development of Sub Saharan African countries. In developed countries, where the prevalence of these infectious diseases is low in comparison to the prevalence in developing countries, blood transfusion centers systematically use the nucleic acid testing (NAT) in the detection of viral pathogens like HIV, HBV, and HCV [11]. By contrast, most blood transfusion centers in Africa continue to use serological techniques in cities and rapid diagnosis testing in rural areas to detect pathogens in blood donation [12]. Beyond the specificity and sensitivity of the PCR, the molecular method is efficient to make a quick and accurate diagnosis. Our study population was made of a large number of non-remunerated young volunteer blood donors and males recruited during social and cultural activities, 196/242 (81.0%). Most of the blood donors (79.8%) were recruited in the city of Ouagadougou and its metropolitan area, and the rest in rural areas (20.2%). Previous studies had indicated that the majority of blood donors in Sub Saharan Africa are young students who live in the big cities [10, 13, 14]. The distribution of prevalence of HbsAg and anti-HCV antibodies in the sampling of our study was respectively 9.9% and 11.6%. The distribution was also higher by 8.7% and 6.3% respectively as found in previous research [15, 16]. Twenty-six (26) cases of RPR + /HCV+ were PCR negative, and 19 cases of RPR + /HbsAg+ from the 94.7% (18/19) sample were PCR positive for the screening of *T. pallidum* DNA, although cases of non venereal treponematosis were not previously included in our screening. The molecular analysis showed the following results: 170/183 (92.9%) and 125/139 (89.9%) respectively positive using serological testing (RPR and CMIA) were negative using PCR. The DNA of *T. pallidum subsp pallidum* bacteria was found in 16/242 (6.6%) of our sampling. Serological tests (RPR, TPHA, CMIA...) do not differentiate a venereal syphilis from other treponemal diseases (yaws or bejel) which are not sexually or congenitally transmitted. However the relevance of the extent of the detection of these pathogens in transfusion lies in the fact that there is a risk of transmission through blood. The rates of sensitivity of serological methods used were 81.3% and 87.5% respectively for the RPR and the CMIA in comparison to PCR (Table 3). In addition, these percentages were in keeping with the recommendations by the Center of Diseases Control (CDC) and World Health Organization (WHO) which are 77-100% for the RPR, 82-100% for CMIA [17, 18].

**Table 3:** Performance of two serological methods using PCR testing as a confirmation test

Tests	Sensitivity	Specificity
RPR	81.3%(13/16)	24.8%(56/226)
CMIA	87.5% (14/16)	44.7%(101/226)

The study found low levels of specificity regarding serologically testing compared to PCR. The explanation resides in the fact that our diagnosis using PCR is based on detection of the specific subspecies that are responsible for venereal syphilis only. Globally, our results showed the efficiency of PCR in the detection of *Treponema pallidum subsp pallidum* while different serological testing is unable to differentiate venereal syphilis from other treponematosis [5, 6]. Due to the high prevalence of transfusion infectious diseases, using a more specific technique such as PCR in the diagnosis of venereal syphilis would help reduce the number of infections contracted during a blood transfusion. Moreover, we noticed that these serological tests may give poor estimates of prevalence because of false positives. The diagnosis is often difficult because of the complexity of the interpretation of results (false negatives). A wrong interpretation of results due to the deficiency of the test used would not only affect the patient, but could also lead to complications or development of congenital syphilis, especially in infants or pregnant women who represent the majority of blood recipients in Burkina Faso.

## Conclusion

The results of the study show the urgent need for extending the molecular diagnosis of pathogens in blood transfusion to *Treponema pallidum*, which will be a more viable alternative to serological testing in the case of poor countries facing a high prevalence of infectious diseases.
